# Nutritional Factors and Therapeutic Interventions in Autism Spectrum Disorder: A Narrative Review

**DOI:** 10.3390/children12020202

**Published:** 2025-02-08

**Authors:** Carlos A. Nogueira-de-Almeida, Liubiana A. de Araújo, Fábio da V. Ued, Andrea A. Contini, Maria E. Nogueira-de-Almeida, Edson Z. Martinez, Ivan S. Ferraz, Luiz A. Del Ciampo, Carla C. J. Nogueira-de-Almeida, Mauro Fisberg

**Affiliations:** 1 Medical Department, Federal University of São Carlos, Brazil—DMED UFSCAR, Rod. Washington Luiz, km 235, São Carlos 13565-905, Brazil; apcontini@ufscar.br; 2 Pediatric Department, Federal University of Minas Gerais, Minas Gerais, UFMG, Avenida Antônio Carlos, 6627, Belo Horizonte 31270-901, Brazil; liubiana@ufmg.br; 3 Department of Health Sciences, Ribeirão Preto Medical School, University of São Paulo, Brazil—FMRP-USP, Av Bandeirantes, 3.900, Ribeirao Preto 14049-900, Brazil; fabioued@usp.br; 4 Nutrition School, University of São Paulo, Brazil—FMRP-USP, Av, Bandeirantes, 3.900, Ribeirao Preto 14049-900, Brazil; menalmeida@hcrp.usp.br (M.E.N.-d.-A.); carlanogueira@usp.br (C.C.J.N.-d.-A.); 5 Department of Social Medicine, Ribeirão Preto Medical School, University of São Paulo, Brazil—FMRP-USP, Av, Bandeirantes, 3.900, Ribeirao Preto 14049-900, Brazil; edson@fmrp.usp.br; 6 Department of Pediatrics, Ribeirão Preto Medical School, University of São Paulo, Brazil—FMRP-USP, Av Bandeirantes, 3.900, Ribeirao Preto 14049-900, Brazil; isferraz@fmrp.usp.br (I.S.F.); delciamp@fmrp.usp.br (L.A.D.C.); 7 Pediatric Department, Federal University of São Paulo, UNIFESP, R. Sena Madureira, 1500, São Paulo 04021-001, Brazil; mauro.fisberg@pensi.org.br; 8 Feeding Dificulties Department, PENSI Institute, PENSI, Av. Angélica, 2.071, São Paulo 01227-200, Brazil

**Keywords:** autism spectrum disorder, food fussiness, feeding and eating disorders, gastrointestinal diseases, microbiota, brain–gut axis

## Abstract

**Objective:** To explore recent findings on how nutritional, gastrointestinal, social, and epigenetic factors interact in autism spectrum disorder, highlighting their implications for clinical management and intervention strategies that could improve development and quality of life of affected children. **Sources**: Studies published from 2000 to 2024 in the PubMed, Web of Science, Scopus, Scielo, Lilacs, and Google Scholar databases were collected. The process for the review adhered to the Search, Appraisal, Synthesis, and Analysis framework. **Summary of the findings**: Children with autism spectrum disorder have restrictive eating habits and often exhibit food selectivity with either hyper- or hypo-sensory characteristics. This review provides an overview of the literature on diagnosis and intervention strategies for selectivity in autism spectrum disorder, including the involvement of family members in meals, sharing a healthy diet and positive relationship with food, and the importance of exploring visual, olfactory, and tactile experiences of food and introducing new foods through play activities to expand the food repertoire. Modifications in the microbiota and gastrointestinal disorders may also be present in autism spectrum disorder and are presented due to their frequent nutritional repercussions. The medium and long-term implications of food preferences and behavior issues for nutritional status are also discussed, given the tendency for children with autism spectrum disorder to consume low-quality and energy-dense foods, leading to nutritional problems. **Conclusions**: Children with autism spectrum disorder have feeding difficulties, especially selectivity, gastrointestinal problems, changes in the microbiota and can evolve with micronutrient deficiencies, malnutrition and obesity. This review describes the evidence for possible targets for interventions aiming to improve nutritional health for children with autism spectrum disorder.

## 1. Introduction

Autism Spectrum Disorder (ASD) is characterized by deficits in communication and social interaction, as well as restricted and repetitive patterns of behavior, interests, or activities [1]. Globally, around 1 in 100 children are affected. At the same time, data from the Centers for Disease Control and Prevention (CDC) in 2023 describe that about 1 in 36 children in the United States is diagnosed with ASD [2]. The scientific literature has been expanding robustly, addressing a range of factors associated with ASD and its comorbidities, including nutritional, environmental, and biological influences that impact the development and health of affected children.

One of the most promising and recent areas of research on ASD involves studies on alterations in the nutritional status and gastrointestinal tract of affected children. Children with autism often exhibit food selectivity, with restricted eating patterns, which can lead to nutritional deficiencies, affecting both physical and cognitive development. Additionally, many individuals with ASD experience gastrointestinal disorders, such as constipation, chronic diarrhea, and gut microbiota-related disturbances, which may exacerbate the behavioral symptoms of the disorder, creating a vicious cycle [3]. Obesity and malnutrition are frequently observed comorbidities, further complicating the clinical picture. Recent research has also focused on the relationship between gut microbiota composition and the neurobehavioral aspects of autism, suggesting that microbial balance may influence brain development [4,5]. This scenario underscores the need for a more integrated approach that considers the multiple dimensions of ASD, particularly those related to the digestive system and nutrition.

Recent advancements in understanding epigenetic mechanisms have shown a significant impact on the development of ASD, elucidating how environmental and genetic factors may interact to influence gene expression and the risk of developing the disorder. Epigenetics, which studies modifications in gene expression without altering the DNA sequence, has become a key area in understanding how factors such as diet and stress during critical periods of development can affect the health and behavior of children with ASD. These findings open new perspectives for preventive and therapeutic interventions based on a more detailed understanding of how the environment interacts with the individual’s biology. Given this complexity, this article reviews recent discoveries on the interactions between nutritional, gastrointestinal, and epigenetic factors in ASD, highlighting the implications for clinical management and intervention strategies that may improve the development and quality of life of affected children.

## 2. Methodology

According to the typology presented by Grant et al. [6], the present article is a literature review including published materials that provide an examination of recent or current literature. The process for the present review adhered to the Search, Appraisal, Synthesis, and Analysis (SALSA) framework: the completeness of searching was determined by scope constraints, there was no formal assessment of the quality of the studies, and a narrative and textual synthesis, as well as a thematic and descriptive analysis, were used. Studies published from 2000 to 2024 in the PubMed, Web of Science, Scopus, Scielo, Lilacs, and Google Scholar databases were collected using the search terms “autism spectrum disorder” or “autism” as the initial basis (n = 32,045). Next, those that addressed the following topics were screened: nutrition, digestive tract, eating behavior, microbiota, dysbiosis, obesity, malnutrition, micronutrient deficiency, wasting, and stunting (n = 342). Finally, review studies and those that did not present clearly defined results were discarded, resulting in 185 studies included. Each of them was read by all authors, who highlighted the most relevant outcomes found. Agreement criteria were defined so that the final text effectively represented the consensus among the members of the research team. The main findings were organized into blocks and are presented in five topics: eating behavior, macro and micronutrients, underweight and obesity, gastrointestinal problems, and microbiota.

## 3. Eating Behavior

The formation of eating habits begins soon after birth, with breastfeeding, and is perpetuated in the life of the human being. This process, which is dynamic and shapes eating behaviors, is built from the combination of genetic, environmental, cultural, socioeconomic, and psychological factors, which will result in future food preferences and choices, subject to modifications throughout life [7]. The existence of several nutritional problems among children diagnosed with ASD is recognized, derived from atypical eating and not-eating behaviors that are very prevalent in this group [8,9,10]. The age of onset can be early while still breastfeeding, with premature weaning already being a possible symptom [11]. However, selectivity and resistance to trying new foods are the symptoms that stand out throughout development [7,12].

Eating behavior is connected to impairments in sensory processing, manifested by excessive responsiveness or atypical responsiveness to sensory stimuli [10,11]. The act of eating involves sensory, emotional, cognitive, and neurological aspects in a complex conjunction of factors. For instance, a study employing a multiple baseline design demonstrated that adding condiments to previously rejected foods, such as vegetables, significantly increased food acceptance across three food items [13]. This finding highlights how sensory enhancements, like condiments, can act as conditioned stimuli, promoting the acceptance of new foods through the establishment of positive food associations [13]. These results suggest that targeted sensory modifications can effectively address food selectivity in children with ASD, facilitating the construction of positive behavioral food decisions. The decision to eat or reject a food goes through sensory perception and processing (visual, olfactory, vestibular, tactile, and gustatory) that, at a responsive level, enables the construction of behavioral food decisions. In ASD, this ability to categorize stimuli, hedonically and cognitively, based on sensory apprehension, seems altered, making it difficult to build models that serve as an internal reference for decision-making at the beginning of the action based on previously acquired experiences. The result of this distorted sensory processing capacity results in discrepant eating behaviors, ranging from total rejection or exclusive preference, with a low number of possible responses [9].

Socialization difficulties in children with ASD often limit opportunities to learn by imitation, a key factor in expanding the food repertoire during childhood [8,11]. Structured interventions, like the MEAL Plan, have shown significant benefits, with a 47.4% positive response rate on the Clinical Global Impression—Improvement scale at week 16, compared to 5.3% for parent education (*p* < 0.05). Additionally, children in the MEAL Plan group consumed 30.76 g more food per meal on average (*p* = 0.001) and showed greater reductions in problematic mealtime behaviors (mean difference of 7.04 points; *p* = 0.01), highlighting its effectiveness in addressing mealtime challenges in ASD [14]. Parents have a significant influence on the development of their children’s eating behavior, so children with ASD in families that choose a healthy diet tend to share such habits [11]. In this aspect, it is difficult for parents to perceive their children beyond the disorder [7], which makes them abandon attempts to stimulate a more regulated food education to give in to their preferences [11].

Eating behavior can be considered to reflect characteristics of ASD defined in the DSM-V: [1] (a) restrictive and repetitive patterns of behavior; (b) insistence on routine; (c) inflexibility; (d) rigid standards; (e) restricted and fixed interests; (f) hyper or hypoactivity to sensory stimuli. Inflexibility arises at mealtimes, manifesting itself in the form of rituals, such as always eating in the same place, the child’s inability to remain seated at the table or difficulty in diversifying the food ingested, making the family environment chaotic and stressful [11] and interfering with adequate nutrient intake [8]. In this scenario, behavioral rigidity is still pointed out as a point of connection between food selectivity and obesity, as it is primarily linked to atypical eating patterns [15,16]. On the other hand, the attempt to ensure adequate intake and sufficient food repertoire for children promotes intense suffering for parents and caregivers of children with ASD [10,12], increasing the likelihood of depression and anxiety symptoms in this group [11].

Selectivity is the most frequently observed profile and refers to a behavior characterized by the frequent or permanent refusal of certain foods or entire food groups due to characteristics such as taste, color, smell, consistency, or form of presentation [17]. This condition is very prevalent in patients with ASD, affecting 50 to 90% of them [18] and being 15 times more common in these children than in their healthy peers [19].

There is a significant variation in the presentation form that can evolve from a very mild condition, which resembles the common food neophobia of childhood, to extreme situations in which no food is accepted [20]. However, an aspect that may accompany this behavior is the presence of an essential disruptive behavior during the meal, almost always mediated by inflexibility [21], that imposes great suffering on the patient and the family [18], including crying, screaming, running away from the dining environment, aggression, spitting, lack of chewing, and throwing food and utensils [22]. Often, the onset of eating difficulties precedes the diagnosis of ASD [23] and other disorders, and these, in addition to selectivity, may be present due to gastrointestinal problems, deliberate restrictions made by parents due to unfounded beliefs, as well as physical and motor difficulties [24]. An additional relevant point is that Avoidant/Restrictive Food Intake Disorder (ARFID) and autism are conditions that often co-occur [25]. In a large Swedish cohort, 12.1% of children with ARFID also had autism, highlighting a strong association between the two conditions [26,27]. Therefore, when evaluating feeding difficulties in children with autism, clinicians should consider the possibility of ARFID, mainly if the child exhibits extreme selectivity or avoidance of foods based on sensory characteristics. In fact, the presence of comorbid neurodevelopmental or psychiatric conditions, such as ASD, ADHD, or anxiety disorders, can increase the likelihood of ARFID [28].

Children with ASD may have food selectivity profiles of hyper- or hypo-sensory characteristics [19,20,29]. The most common aspect is related to hyper-sensoriality [3], leading to refusal due to low tolerance to environmental stimuli [17]. Almost always, the condition exceeds the limits of food, and intolerant behaviors are observed in relation to multiple aspects of daily life, such as noise, odors, and light, among others [23]. These children do not eat well because they feel the characteristics of food very intensified [19] and may resemble “supertasters” [30]. We suggest calling this behavior **“hyper-sensory selectivity”.** However, in some cases, the opposite aspect is observed, and some patients, due to ASD, have great difficulty in performing the central processing of information from the environment [31]. In this way, it is as if they were less likely to perceive flavors, smells, colors, noises, and textures, which configures a hypo-sensoriality profile [3]. These children do not eat well because they do not feel the food [19]. We suggest calling this profile **“hypo-sensory selectivity”.** The differentiation between the two types is relatively simple. When it comes to hyper-sensoriality, refused foods, when offered, generate repulsion, nausea, and intense discomfort for the child [23]. In cases of hypo-sensoriality, there is an evident lack of interest in everyday foods, which are refused due to the fact that they do not cause any interest in being ingested, and there may be better acceptance of foods that are sweet, salty, fatty and highly palatable [20]. In both cases, the severity of symptoms is variable and may be pretty low, with little or no repercussions on nutritional status. On the other hand, relevant consequences are often observed, which can lead to severe malnutrition, evolving to intense thinness, stunting, hidden hunger, or obesity [3]. These different outcomes may be a consequence of energy insufficiency, leading to negative caloric balance in extreme selectivity; of the small variety, leading to vitamin and mineral deficiency; or compulsive and repetitive eating profiles in which few foods are consumed in large quantities, with a frankly positive energy balance. Changes in nutritional status will be detailed later.

Considering the therapeutic approach of selectivity in a generic way, supplementation with complete and isocaloric profile supplements has been used with satisfactory results to ensure the adequate supply of macro- and micronutrients [32,33,34,35,36,37]. So far, there is no consensus on how to treat selectivity linked to ASD [38], but some strategies have been adopted and described in the literature. The recommendation is that, whenever possible, the approach should be multidisciplinary [38].

In cases of hypo-sensory selectivity, the child may sometimes respond to hyperstimulation [13]. This can be achieved by increasing the sensory properties of food, making it, for example, crunchier, more colorful, sweet, salty, seasoned, spicy, noisy, and fragrant [30,33]. Substances such as curry, peppers, herbs, and colorants can be used to modify foods, making them capable of producing more intense sensations and more likely to be perceived, processed, and interpreted in the central nervous system [33]. One of the problems for treatment in these cases refers to the fact that acceptance may be better for highly palatable foods that, in general, are richer in carbohydrates, lipids, salt, and sugar. This, in association with the other characteristics of ASD, especially tendencies to impulsivity, can increase the risk of progression to obesity, insulin resistance, hypertension, and dyslipidemia [20]. Hyper-sensory selectivity, on the other hand, has a more complex treatment. From the point of view of food, the trend is a greater acceptance of those with a more neutral profile, less colorful, less striking flavor and odor, and liquid or pasty consistency [33].

Due to the refusal behavior associated with phenomena such as nausea, vomiting, choking, and neurovegetative disorders, the moment of the meal becomes challenging. For this reason, the therapeutic process must involve the whole family [23]. The participation of parents in meals should be encouraged and characterized as fundamental [7], because they are the ones who will serve as models and will be the promoters of the interventions. The environment has a substantial impact on eating habits; for this reason, it is recommended that the family share a healthy diet, establishing a positive relationship with food at mealtime [11,39]. Changes in the family’s lifestyle are necessary, based on adequate nutritional guidance [10]. Adjustments must be made to the food profile and the environment where meals are held, as well as the preparation of a schedule that meets the difficulties and demands in an individualized way, respecting the needs and uniqueness of each case [10]. Guidance for the expansion of sensory aspects, with the offer of new foods and the inclusion of other forms of experiences that involve the purchase and preparation of food, are resources to be used [7]. Exploring visual, olfactory, and tactile experiences of food in the form of actions that increase sensory familiarization allows the development of the hedonic characteristics of sensory stimuli, resulting in the expansion of the food repertoire in children with ASD [9]. Toomey and Ross (2011) advise that children with food selectivity are slowly introduced to food in progressive steps through the use of a “desensitization” hierarchy to encourage them to explore, interact, and eventually eat new foods. This hierarchy involves six main categories: “tolerating”, “interacting”, “smelling”, “touching”, “tasting”, and “eating” the foods presented and is used to encourage sensory processing and the acquisition of oral motor skills [40]. This hierarchical scale was called the Sequential Oral Sensory Approach to Feeding (SOS Approach) [40] and appears to be beneficial for children with neurological impairment, including autism [41]. In general, initially, the professional must understand which sensory changes (tactile, olfactory, visual, auditory, and/or gustatory sensitivity) impact the child’s diet. Then, familiarization with new foods will begin through playful activities involving sight, smell, and touch. Then, the gradual advancement of taste and contact with food will be achieved through a series of small steps [34]. By respecting the child’s sensory processing, there is a greater probability of approaching the food. Professionals to be involved in the interdisciplinary management of food selectivity include occupational therapists, nutritionists, psychologists, speech therapists, and physicians [42]. Regarding ARFID, some authors highlights cognitive behavioral therapy (CBT) as a possible intervention [43,44]. CBT can be performed in conjunction with sensory integration or “desensitization” methods [34]. This means that the introduction of new foods can be done in stages or phases, setting sensory goals to “desensitize” the child and subsequently ensure familiarity with the taste and texture of the food [45,46].

**Figure 1:**
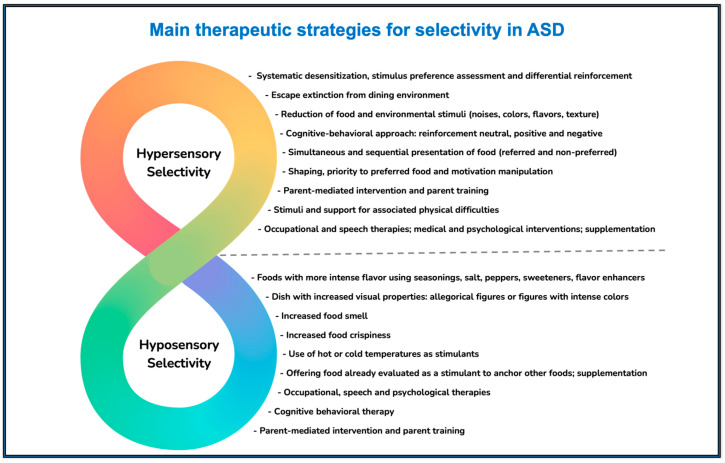
Main therapeutic strategies for selectivity in ASD.

The main therapeutic strategies for selectivity in ASD described in the literature are summarized in Figure 1 [37,47,48,49,50,51,52,53,54,55,56,57,58,59,60,61,62,63,64].

## 4. Macro and Micronutrients

The food preferences of children with ASD [65] contribute to increasing the difficulty in composing meals and introducing new foods, resulting in preferences for refined, processed, soft, and sweet foods [10,66,67] and the rejection of vegetables, fruits, and grains, along with sour and bitter flavors [66,68,69]. Limitations in food categories are observed [68,70], associating altered and inflexible rituals and behaviors [10,66,71] with compromised quality and quantity of nutrients in the diet, leading to medium- and long-term repercussions on nutritional status [66,72,73].

The increased consumption of energy-dense foods with high amounts of carbohydrates [67,68] and fats [74] can lead to excessive weight gain. Studies conducted on all continents have found a high prevalence of obesity [10,42,66,74,75,76,77,78]. On the other hand, protein intake falls short of requirements since the primary sources of this nutrient are scarce in these children’s diets [79,80,81,82].

The association of frequently observed characteristics, sometimes in an extreme form [83], such as oral defensiveness, food selectivity, neophobia, and gastrointestinal disorders [71,73], contributes to an increased risk of deficiency of several micronutrients [8,65,84,85,86]. Regarding minerals, low ferritin concentrations have been observed in children with ASD, mainly due to the low intake of meat, whose appearance and texture are usually rejected [76,81,87]. This leads to an increased risk of anemia, contributing to reduced cognitive performance and accentuating some characteristics of ASD itself [53,85,88]. Zinc deficiency is also common among children with ASD and may contribute to impaired neurodevelopment and immunity [25,68,80,88,89,90,91]. Changes in appetite and taste associated with low zinc concentrations contribute to increased eating difficulties [92]. Calcium has also been found in low concentrations among children with ASD [68,69,79,80,86,93]. Chronic calcium deficiency can result in long-term problems, especially osteopenia and osteoporosis [76,81].

Patients with ASD are also at higher risk of vitamin deficiencies [12,69]. Vitamin D deficiency (VDD) has been frequently reported [94,95,96,97], and lower serum concentrations of 25-OH-vitamin D are observed in this group [95,97]. Low intake [69,79,98], genetic factors [99], and reduced sun exposure (due to difficulties with social interaction and subsequent isolation) contribute to this condition [100]. Obesity, often associated with ASD [101], is another risk factor [102]. At the same time, VDD may exacerbate symptoms associated with ASD, which are alleviated after the correction of the deficiency [103], as observed in studies in China and Turkey [104,105]. An “inflammatory state” in specific brain areas could help explain these findings [106,107], as there is a negative correlation between serum concentrations of 25-OH-vitamin D3 and peripheral inflammation markers [108]. Regarding vitamin A, although some studies have reported serum retinol concentrations within the normal range in children with ASD [109,110], cases of vitamin A deficiency (VAD), sometimes severe, with ocular symptoms of deficiency (night blindness, xerophthalmia and loss of vision) have been reported [111,112]. Godfrey et al. reported the cases of six individuals with VAD, impaired visual acuity, and improvement following vitamin A supplementation [113]. The ocular manifestations of severe VAD may be aggravated by hyperostosis of the optic canal, with consequent compression of the optic nerve [114]. A Chinese study with children aged two to seven observed, among boys, a negative correlation between serum retinol concentrations and scores on the Social Responsiveness Scale and the communication warning behavior subscale of the Children Neuropsychological and Behavior Scale (2016 Revision) [110]. Regarding vitamin C, the development of scurvy in children with ASD was reported by Sharp et al. [115] and Kinlin and Weinstein [116]. Some studies have also found a lower intake of B-complex vitamins among individuals with ASD [117,118], and several authors have highlighted vitamin B12 (cyanocobalamin) as frequently deficient in these cases [117,119,120,121]. Additionally, some studies have reported deficient serum concentrations of vitamin B9 (folate) [104,117,121] Deficiencies in vitamin B6 (pyridoxine) and vitamin B1 (thiamine) have also been reported [104,122].

Given the dietary profile that includes imbalance or deficiency in the intake of macro- and/or micronutrients, the initial treatment should be readjusting the diet. However, given the characteristics of these children, this measure presents varying degrees of difficulty and takes time to produce the desired effects. Broader nutritional supplementation, through complete supplements, has been adopted as an alternative [32,33,34,35,36,37]. Supplementation should be initiated gradually, using products that provide general benefits with minimal side effects, always under the supervision of a healthcare professional [72,123]. However, there is no consensus regarding dosages and duration of use [124]. Amino acid supplements may help regulate neurotransmitters in the central nervous system [125]. Several authors have demonstrated that DHA supplementation contributes to improvements in social communication, behavior, and cognition. Minerals such as iron, zinc, and magnesium have also been used successfully and have contributed to improvements in sleep conditions [87], cognitive performance, and motor skills [126,127]. The treatment of vitamin and mineral deficiencies is outlined in Table 1 [47,48,49,50,51,52,53,54,55,56,57,58,59,60,61,62,63,64].

## 5. Underweight and Obesity

Individuals with ASD face unique challenges in relation to their nutritional status and are at greater risk of being underweight, although the prevalence of overweight and obesity is also significant. This dual risk, which can also be associated with micronutrient deficiencies, highlights the complexity of nutritional problems in ASD, requiring careful monitoring and personalized interventions [128]. Children with ASD have a 6.5% prevalence of being underweight and a 28.5% higher risk of being underweight compared to neurotypical controls [129]. Malhi et al. observed lower levels of certain micronutrients and lower growth parameters in children with severe ASD, who were smaller and lighter compared to those with less severe symptoms [130]. The relationship between ASD and body weight is complicated by the presence of other health conditions in which present and past comorbidities can influence nutritional status [131]. The association with psychiatric conditions such as anorexia nervosa suggests that underweight may be exacerbated by restrictive eating behaviors, although this relationship is complex and not fully understood [132]. Nutritional deficit is a pattern in children with greater severity of the spectrum, and the greater the inflexibility and food restriction, the more significant the low weight. A study by Bölte et al. showed that 28% of males with ASD had a body mass index (BMI) in the fifth percentile or below, suggesting a significant presence of low body weight in this population. Still, this association was inconsistent and partially mediated by hyperactivity [133]. According to Bölte et al., maladaptive social and communicative behaviors, as well as stereotypical characteristics, do not show a significant association with BMI, except for hyperactive behavior, which was partially responsible for low body weight in some cases [133]. ARFID, on the other hand, when associated with ASD, can often lead to low weight [26,27].

A population-based study in Israel showed that low birth weight and premature birth were associated with a higher risk of ASD, potentially affecting growth trajectories [134]. A Chinese study has shown that overall body growth may not be so significantly affected in early childhood [135].

Although the relationship between ASD and low weight or height is clear, it is important to consider the variability within the affected population. Factors such as symptom severity, eating behaviors, and developmental problems play crucial roles in influencing growth. More research is needed to understand these dynamics better and develop targeted interventions that meet the specific needs of individuals with ASD. Treatment will depend on food intake capacity, the degree of selectivity, and the severity of the condition. Children with a higher degree of involvement have greater inflexibility and difficulty in accepting foods with nutrients that are in deficit. More significant dietary restrictions lead to greater risks of malnutrition and an association with ARFID. Nutritional planning should consider hypercaloric supplements (up to 1.5 Cal/mL) if there is milk acceptance. The possibility of using powdered supplements with food will be considered if there is minimal acceptance of staple foods. In cases of extremely low weight, intense selectivity for a prolonged period, unsuccessful oral acceptance, and lack of response to conventional interventions, preferential gastric feeding should be considered, with gastrostomy and a gastric inlet button for the shortest possible time [72,136,137,138].

Childhood obesity is a growing public health problem with a negative impact on physical and mental health. It is associated with comorbidities such as type 2 diabetes, dyslipidemia, non-alcoholic fatty liver disease, and cardiovascular diseases, as well as social and emotional consequences such as bullying and depression [139]. Children and adolescents with ASD have a higher prevalence of obesity compared to healthy controls [140,141]. Systematic reviews with meta-analysis estimate that this prevalence varies between 7.9% and 31.8% [78,142]. The relative risk of obesity in children with ASD ranges from 1.41 (95% CI: 1.062–1.876) to 1.58 (95% CI: 1.34–1.86) [78,142]. The reasons for the development of obesity in children with ASD are multifactorial [143], and many of them are similar or additional to the causes already defined for the general population [141]. Maternal metabolic disorders during pregnancy, such as diabetes, hypertension, and obesity [143], and shorter duration of exclusive breastfeeding are also associated with a higher risk [144,145]. Food selectivity contributes to a preference for energy-rich foods, sweetened beverages and snacks [146], and a low intake of vegetables [146,147]. The use of medications, such as second-generation antipsychotics, to reduce disruptive behaviors also contributes to substantial weight gain [148,149]. Children with ASD spend less time engaged in physical activities [150] due to social and behavioral challenges [151], motor deficits [152], and increased screen time [153,154]. Poor sleep quality is also associated with weight gain [155,156]. Other risk factors involve changes in specific appetite hormones, such as leptin, adiponectin, and ghrelin, as well as changes in the intestinal microbiota, which are more prevalent in ASD and individuals with obesity [143]. Finally, genetic vulnerabilities, such as deletions in 16p11.2, have been associated with obesity and ASD [157,158].

Health professionals must work on preventing obesity in ASD to minimize the risk of associated comorbidities, which can considerably worsen the child’s quality of life. Once the diagnosis of obesity is confirmed, the initial (and first-line) treatment is similar to that for neurotypical children and involves behavioral and dietary modifications. However, these first therapeutic steps can be problematic for children who have difficulties with social and behavioral communication, difficulties with changes in routine, sensory processing, and decision-making [159,160]. Despite this, efforts must be concentrated on improving the consumption of fruits, vegetables, and legumes, which are capable of reducing the energy density of the diet, being sources of dietary fiber and impacting the development of the microbiota; reducing the consumption of sugary drinks and high-calorie foods; encourage physical activity [161]; involve the whole family in changing eating habits and physical activity [162]; refer to physiotherapy sessions in case of motor difficulties [163]; in addition to reducing screen time and improving sleep quality, establishing bedtime routines [45]. Replacing obesogenic drugs, frequently prescribed in ASD, with others without this effect can be discussed with a neurologist or psychiatrist [164]. GLP1 agonists have not yet been sufficiently studied in ASD. Still, their use is authorized for typical children from 12 years of age, configuring a therapeutic option to be considered, especially when binge eating is present [165].

## 6. Gastrointestinal Issues

ASD is associated with a variety of gastrointestinal (GI) disorders that can be generated or influenced by aspects related to social interactions, communication difficulties, repetitive movements, behavioral changes (irritability and aggression), and psychiatric disorders [166]. Food preferences and selectivity exacerbate gastrointestinal symptoms [18]. The prevalence of GI problems in individuals with ASD is considerably high and more prevalent in these individuals than in those with other causes of developmental delays [167], ranging from 46% to 84%, depending on the assessment method and population studied [73,168]. GI issues in individuals with ASD significantly impact the quality of life of children and their families, affecting well-being, school attendance, and participation in social activities [169]. The main clinical manifestations are constipation, chronic diarrhea, abdominal pain, nausea, abdominal distension, and gastroesophageal reflux [170], which contribute to the worsening of the behavioral and sensorimotor manifestations in affected children [171]. This interference suggests complex interactions involving the brain–gut axis [172]. Factors related to genetic mutations and variations [172], altered gut microbiota [166], as well as food intolerances and allergies, particularly to gluten and casein [173], are implicated in gastrointestinal manifestations. Immune system dysfunction is observed in some children with ASD [172]. Chronic intestinal inflammation, altered gut motility, and increased intestinal permeability are considered relevant mechanisms in the manifestations of GI issues [174]. 

Ferguson et al. (2019) investigated GI issues in 340 children and adolescents with ASD and found constipation to be the most prevalent symptom (65% of cases), followed by stomach pain (47.9%), nausea (23.2%), and diarrhea (29.7%). In this study, the presence of aggressive behavior was associated with nausea in children aged 2 to 5 years, and among those aged 6 to 18 years, anxiety behavior increased by 11% in the presence of constipation symptoms. The association of differences in sensory processing and integration may exacerbate the response to gastrointestinal symptoms and discomforts [175].

The treatment for GI disorders varies and is related to individualized approaches tailored to each specific manifestation, considering the complexity of each patient. The modulation of gut microbiota through prebiotics and probiotics is discussed in this article. Studies investigating dietary interventions, such as the elimination of gluten and casein, the reduction of complex carbohydrate, lactose, and sucrose intake, and ketogenic diets, among others, have shown inconsistent and potentially harmful results due to the lack of scientific evidence on its effectiveness, to the strict adherence required for these diets and to the risks of nutritional deficiencies [124,176]. An individualized dietary approach is essential to meet the specific needs of everyone with ASD and assist in the treatment of their symptoms. For constipation, treatment generally includes the use of laxatives (such as polyethylene glycol) to facilitate bowel movements and relieve discomfort. Dietary adjustments are recommended, such as paying attention to food preferences, including or adjusting fiber intake, encouraging fluid intake, establishing a bathroom routine after meals, and promoting regular physical exercise [73,177]. It is important to remember that constipation can also be associated with sensory issues related to contact with feces or odors, as well as vestibular limitations that can make sitting on the toilet complex. These aspects should be considered for an appropriate intervention.

Chronic diarrhea’s higher occurrence may be related to sensory behavior (preferences/aversions), food sensitivities/intolerances, side effects of medications used to improve ASD-related symptoms, anxiety, and stress. Treatment aims to address the cause of diarrhea. It should be individualized, enable hydration, and re-establish a balanced diet adjusted to the child’s nutritional needs. Recognizing the cause and having strategies that facilitate adherence to the treatment are vital for controlling the diarrheal process [73,171].

Abdominal pain impacts the quality of life of children with ASD, and the difficulties in understanding painful events and communicating discomfort lead to problems in proper diagnosis and treatment [167,178]. Painful sensations can intensify aggressive behaviors, abnormal vocalizations, motor signs, hyperactivity, anxiety, and alter sleep patterns [175]. The treatment should focus on the cause of abdominal pain (constipation, GER, irritable bowel syndrome, food intolerances) and sensory, emotional, medicinal, musculoskeletal, and urinary factors. The approach must be comprehensive, collaborative, and individualized, with dietary modifications, behavioral therapies, and prescription medications for symptom relief. A multidisciplinary approach involving pediatricians, gastroenterologists, and behavioral therapists is desirable. The appropriate treatment, tailored to specific needs, will improve the quality of life and overall well-being of patients with GI disorders and ASD.

## 7. Microbiota

The gut microbiota regulates hormonal and inflammatory functions, which directly impact digestive function and the nervous system through the brain–gut axis [179]. Thus, an unsatisfactory microbiota composition can compromise the intestinal epithelial barrier, increase intestinal permeability, and alter the synthesis and release of neurotransmitters (serotonin, gamma-aminobutyric acid, and oxytocin) [179].

The influence of the gut microbiota in patients with ASD has been studied since the 1990s when it was observed that the use of antibiotics, initially vancomycin, resulted in behavioral improvements and gastrointestinal symptoms in these patients [166]. Recent studies have shown that people with ASD have significant changes in their gut microbiota [180]. Concomitantly, chronic gastrointestinal alterations are found in up to 70% of children with ASD and can manifest in the forms of diarrhea, constipation, gastroesophageal reflux, alteration of the permeability of the intestinal barrier, immune dysregulation, and inflammation of the gastrointestinal tract [180,181].

Several factors seem to contribute to these changes, such as type of delivery, gestational age, genetic factors, and diet, in addition to the use of antibiotics, which are more frequently prescribed to ASD patients [180,181,182]. Studies suggest that the microbiota of these individuals has a lower diversity of microorganisms, high levels of *Clostridium*, *Bacteroides*, and *Desulfovibrio*, and lower levels of *Actinomyces* and *Firmicutes*, which correlates with the severity of gastrointestinal manifestations, with higher production of short-chain fatty acids, especially propionic acid, which has been related to the development and severity of the ASD symptoms [179,180,181]. When food selectivity is present, high values of *Prevotella*, *Anaerophilum*, *Clostridium*, and *Salmonella are observed*, in addition to a higher *Escherichia*/*Shigella ratio* [82].

There is a large discrepancy between microbiota profiles, which is attributed to the variables gender, age, previous comorbidities, use of medications, use of antibiotics, diet, cultural differences, and environment [181,182,183]. Thus, it is not possible to establish a pattern of varieties and concentrations of microorganisms in the microbiota of the population with ASD, so a generalized treatment cannot be determined. Studies suggest that the use of probiotics can improve gut dysbiosis, which would result in modulation of the immune system, attenuation of gastrointestinal symptoms, reinforcement of intestinal barrier function, and regulation of the production of neuroactive components [171]. Gut microbiota is involved in modulating neurotransmitters like dopamine, GABA, and glutamate, which have implications for neuropsychological disorders and gastrointestinal diseases [184].

In a recent review, Mhanna et al. state that targeting the gut microbiome represents a promising intervention for patients with ASD [184]. Still, studies present controversial and uncertain results about the treatment protocols and actual benefits of this supplementation [171,181]. Researchers attribute these limitations to the significant variability in the strains, varied methodologies, small sample sizes and the and the interference of environmental factors [181]. The strains that seem to show the best results are Lactobacillus and Bifidobacterium, that could possibly improve dysbiosis, reduce gut inflammation, increase Bacteroidetes/Firmicutes ratio and improve behavioral symptoms [171,181]. According to a systematic review published in 2024 by Al-Beltagi et al., probiotics supplementation can be used as an adjunct in the management of ASD symptoms, especially when GI issues are noticed, considering practical aspects as the choice of strains, the appropriate clinical protocol, the safety of the samples and the individual response to the treatment [171].

Prebiotics, in turn, promote an increase in populations of beneficial microorganisms, which can impact intestinal health, improve stool consistency, and reduce inflammation of the tract. In animal models, it is suggested that the modulation of the immune system resulting from supplementation with prebiotics culminates in positive effects on the behavior and cognition of individuals with ASD [171]. However, studies in humans are scarce, and standardized doses of supplementation have not yet been established, although it is recommended to include foods that are sources of prebiotics (onions, garlic, bananas, asparagus, among others) in the diet of these patients, adding to a healthy and balanced dietary context [171,181].

Another treatment that has been studied is fecal microbiota transplantation, which appears to result in more prolonged effects on chronic intestinal disorders and has actions to reduce symptoms of anxiety and depression [181]. On the other hand, there is a greater risk of adverse effects, in addition to being an experimental, invasive, and discouraging procedure, especially in this population [181].

## 8. Limitations

We decided not to conduct a systematic review with meta-analysis due to the heterogeneity in the scientific literature, particularly regarding the lack of standardization in the diagnostic criteria for ASD and its comorbidities, as well as the methodological variations in the nutritional interventions described. These variations include differences in intervention duration, the nutrients targeted for inclusion or exclusion, the protocols associated with behavioral modification, among other factors. For this reason, it was decided to carry out a narrative review, through articles originating only in journals indexed in the main databases. Thus, an important limitation of the present study refers to the fact that it is not possible to generalize the information obtained, which cannot be taken as absolute truths, but rather as paths for intervention opportunities and future investigations. As is inherent to literature reviews, this manuscript has some limitations, among which the bias of potential omission of sections of the scientific literature and/or for not questioning the validity of the statements made in the reviewed articles [6]. Therefore, whenever possible, this paper integrated the statements of the reviewed articles, although recognizing that this was not always possible due to the great heterogeneity of the scientific literature, this aspect being another limitation of this review.

The paper highlights the lack of consensus regarding dosages and duration of nutritional supplementation for children with Autism Spectrum Disorder (ASD), which complicates treatment approaches. It notes the difficulty in adjusting diets due to the restrictive eating habits of children with ASD, leading to challenges in introducing new foods. The variability in individual responses to dietary interventions and the influence of environmental factors on gut microbiota complicate the establishment of generalized treatment protocols. Additionally, the paper mentions that many signs and symptoms associated with ASD can also be found in the general population, complicating diagnosis.

## 9. Conclusions

**Figure 2:**
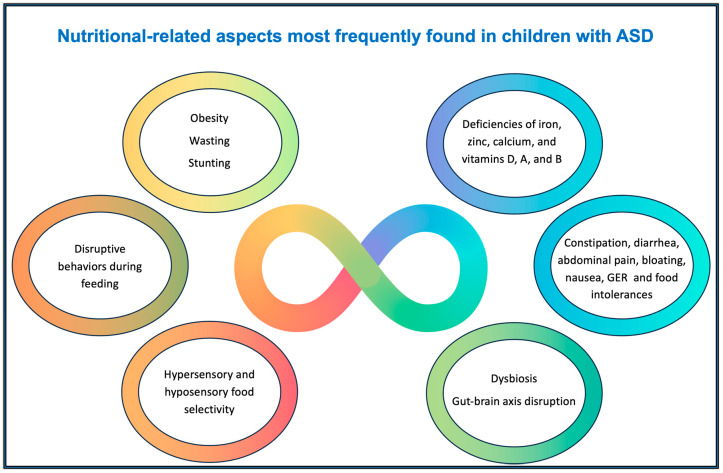
Nutritional-related aspects most frequently found in children with ASD.

Many children with ASD have restrictive eating habits, leading to nutritional deficiencies that affect physical and cognitive development. They also often experience gastrointestinal problems such as constipation, diarrhea, and gut microbiota imbalances, which can exacerbate behavioral symptoms. This review explored recent evidence on how nutritional, gastrointestinal, and epigenetic factors interact in ASD, highlighting their implications for clinical management and intervention strategies that could improve the development and quality of life of affected children. The diversity of eating habits, defining different profiles of food selectivity, highlights that strategy to provide adequate nutrition for children with ASD cannot be based on a single approach but must consider their personal preferences and different ways of dealing with sensory aspects. It also emphasizes that the therapeutic process must include the participation of the child’s whole family in promoting healthy eating habits and that interventions for the practical approach to food is a challenge that requires the involvement of a multidisciplinary professional team. It is important to note that many of the signs and symptoms described in the present review are not exclusive to ASD and can be found in the general population and may even be present in children with ASD without a cause-and-consequence relationship. Figure 2 seeks to summarize the nutritional-related aspects most frequently found in children with ASD.

## Figures and Tables

**Table 1:** Treatment of vitamin and mineral deficiencies.

Micronutrient	Therapeutic Scheme
Iron	Oral dose of 2 mg/kg/day of elemental iron, for a period sufficient to recovery of ferritin and hemoglobin
Magnesium	Oral dose of 6 mg/kg/day when serum values are below 1.7 mg/dL or when the dietary survey shows evident deficiency in consumption
Zinc	Oral dose of 2 mg/kg/day of elemental zinc, respecting the maximum dose of 20 g/day, until serum zinc concentration reaches 70 mg/dL
Calcium	Oral dose of 100 mg per day of elemental calcium while the nutritional risk situation persists
Vitamin D	Oral dose for 90 days: - Under 1 year of age: 2000 IU/day - Between 1 and 12 years of age: 3000 to 6000 IU/day - Over 12 years of age: 6000 IU/day
Vitamin A	Oral dose of retinol palmitate: - Under 6 months of age: 50,000 IU/day - Between 6 months and 1 year: 100,000 IU/day - Over 1 year (male): 200,000 IU/day - Between 1 and 12 years (female): 200,000 IU/day - Over 12 years (female): 10,000 IU/day or 25,000 IU/week for three months in cases of night blindness and/or Bitot’s spots - Note 1: Treatment should only be administered to individuals with clinical manifestations of VAD (xerophthalmia) or severe malnutrition (regardless of the presence of vitamin deficiency) - Note 2: Treatment will be in a single dose for cases of severe malnutrition and in three doses (D1, D2 and D14) in cases of xerophthalmia and/or active lesions (ulcers) in the cornea - Note 3: In cases of pregnant adolescents or those suspected of being pregnant, the initiation of treatment for active corneal lesions (considered an emergency due to the risk of vision loss within 24 to 48 h) should be carefully weighed against the risk of maternal blindness and the potential undesirable effects of vitamin A on the fetus. Administration of vitamin A to women of childbearing age should be done with extreme caution due to the risk of teratogenicity to the fetus
Vitamin C	Oral dose of 100–300 mg/day of vitamin C for infants and children for one month or until full recovery occur
Vitamin B12	- Initial dose: 0.2 μg/kg, subcutaneously for two days (attention to the possible hypokalemia during this phase in children with severe anemia) - After the initial dose: 1000 μg/day, subcutaneously for 2 to 7 days; subsequently, a dose of 100 μg/week, subcutaneously, for one month
Folate	Oral dose of 1 to 5 mg/day of folic acid for up to four months; in infants, doses of 50 µg/day may be sufficient
Vitamin B6	Except for cases of children with pyridoxine-dependent syndromes who present with seizures, the doses for vitamin B6 supplementation in the pediatric population in cases of nutritional deficiency are not well defined

## Data Availability

Not applicable.
